# Aged care residents’ prioritization of care: A mixed‐methods study

**DOI:** 10.1111/hex.13195

**Published:** 2021-01-21

**Authors:** Kristiana Ludlow, Kate Churruca, Virginia Mumford, Louise A. Ellis, Jeffrey Braithwaite

**Affiliations:** ^1^ Australian Institute of Health Innovation Macquarie University Sydney NSW Australia

**Keywords:** aged care, decision making, nursing homes, person‐centred care, priority‐setting, residential facilities

## Abstract

**Background:**

Eliciting residents’ priorities for their care is fundamental to delivering person‐centred care in residential aged care facilities (RACFs). Prioritization involves ordering different aspects of care in relation to one another by level of importance. By understanding residents’ priorities, care can be tailored to residents’ needs while considering practical limitations of RACFs.

**Objectives:**

To investigate aged care residents’ prioritization of care.

**Design:**

A mixed‐methods study comprising Q methodology and qualitative methods.

**Setting and participants:**

Thirty‐eight residents living in one of five Australian RACFs.

**Method:**

Participants completed a card–sorting activity using Q methodology in which they ordered 34 aspects of care on a pre‐defined grid by level of importance. Data were analysed using inverted factor analysis to identify factors representing shared viewpoints. Participants also completed a think‐aloud task, demographic questionnaire, post‐sorting interview and semi‐structured interview. Inductive content analysis of qualitative data was conducted to interpret shared viewpoints and to identify influences on prioritization decision making.

**Results:**

Four viewpoints on care prioritization were identified through Q methodology: Maintaining a sense of spirituality and self in residential care; information sharing and family involvement; self‐reliance; and timely access to staff member support. Across the participant sample, residents prioritized being treated with respect, the management of medical conditions, and their independence. Inductive content analysis revealed four influences on prioritization decisions: level of dependency; dynamic needs; indifference; and availability of staff.

**Conclusions:**

Recommendations for providing care that align with residents’ priorities include establishing open communication channels with residents, supporting residents’ independence and enforcing safer staffing ratios.

## 1 INTRODUCTION

### 1.1 Person‐centred care

In shifting towards more person‐centred approaches to care, service user involvement is increasingly recognized as an essential part of health‐care provision.^1^ One of the core elements of person‐centred care is acknowledging and respecting service users’ preferences.^2^ Ensuring that service users receive person‐centred care is particularly important in residential aged care facilities (RACFs) as context‐related barriers have the potential to limit residents’ involvement in their care. These barriers include organizational factors such as task‐oriented care and rigid routines,^3^ resident characteristics including cognitive impairment, communication problems and dependency on others,^4^, ^5^ and factors associated with the transition into residential living (eg loss of autonomy).^6^ Seeking out residents’ preferences for their care is a necessary, albeit sometimes challenging, process in facilitating person‐centred care.

### 1.2 Preferences and prioritization

Self‐report tools such as the Preferences for Everyday Living Inventory for NH residents (PELI‐NH),^7^, ^8^ the Resident VIEW^9^ and the Minimum Data Set 3.0 Preference Assessment Tool (MDS‐PAT)^10^, ^11^ have been used to elicit residents’ care‐related preferences. These types of assessments require residents to rate domains of care by level of importance with no restrictions placed on rankings, that is, residents can rank every item at the highest level of importance. This is a potential limitation of preference assessment tools, as they do not adequately account for the complex, resource‐constrained and often pressurized environments of RACFs.^3^, ^12^

Assessing residents’ priorities can overcome this limitation. Prioritization of care, by definition, requires determinations about the *relative* importance of different aspects of care, in light of, for example, environment, circumstances and the availability of resources. In health‐care services literature, prioritization refers to ordering care tasks by levels of importance or urgency when available resources are inadequate.^13^, ^14^ Although prioritization is primarily associated with health‐care workers’ delivery of care, it is also a relevant concept for resident populations in terms of establishing and understanding their priorities for their care.

### 1.3 Rationale

Studies of care prioritization in RACFs have predominately focused on health‐care workers’ perspectives,^15^ and therefore, a knowledge gap persists regarding residents’ views. By understanding what and how residents prioritize, policymakers, aged care providers and front‐line staff can target improvement efforts to better align care with residents’ needs and expectations.

### 1.4 Objectives

The objective of this study was to investigate aged care residents’ prioritization of care. The study had three research questions:

1. What are residents’ priorities regarding their care?
2. How do residents prioritize care?
3. What influences aged care residents’ prioritization decisions?

## 2 METHODS

### 2.1 Study design

The research comprised a mixed‐method multi‐site study involving Q methodology and qualitative methods. It is part of a larger research project exploring the prioritization of care in RACFs.^16^

### 2.2 Sample and setting

Participants were residents living at one of five participating RACFs located in the Australian States of Queensland and New South Wales. The facilities were managed by a single aged care provider. Purposive sampling, a common convention of Q methodology, was used to recruit participants. Recruitment was guided by the following inclusion criteria: willingness and ability to provide informed consent; capacity to participate in an English‐language interview; and participation in the study would be unlikely to be burdensome for residents (physically and/or emotionally). Facility managers, care managers and designated staff members identified residents who met the inclusion criteria. Participants were invited to participate in the study through invitation letters delivered face‐to‐face by the lead researcher or a member of staff. The invitation letters explained that the research formed part of KL’s doctoral studies.

### 2.3 Materials

**FIGURE 1:**
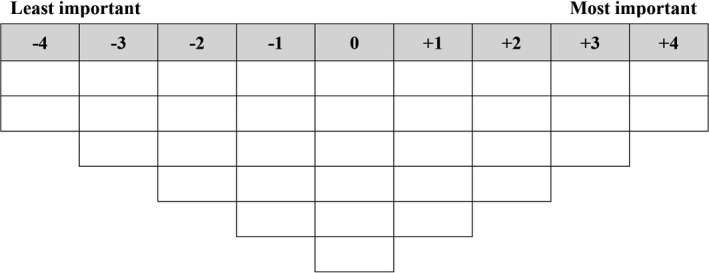
Q sort grid

Materials for the card‐sorting activity comprised a set of 34 cards (Q sort deck), each representing an aspect of care, and a forced distribution sorting grid (Q sort Grid) on which participants ordered the cards (Figure 1).^17^ The Q sort deck was taken from our related studies of staff and family members’ prioritization of care,^16^, ^18^ with slight modifications. For example, ‘residents have independence’ and ‘my family member has independence’ were modified to ‘I have independence’. Other study materials included a basic demographic questionnaire, post‐sorting interview questions and a semi‐structured interview guide. Post‐sorting interviews asked participants about the placement of salient cards and about additional care activities not represented by the Q sort deck. Semi‐structured interviews covered the following topics: communication of priorities, the priorities of other stakeholders and unmet priorities.

### 2.4 Data collection procedure

Participants were first asked to sort the Q sort deck into three piles with regards to their care preferences: Least/less important, Neutral/somewhat important and Most/more important. They used these piles to order the cards on the Q sort grid from ‘Least important’ (−4) to ‘Most important’ (+4) in terms of the care they received. During this activity, participants verbalized their decision making through a think‐aloud task.^19^, ^20^ Upon completion of the card‐sorting activity, participants were given the opportunity to adjust their card‐sorting pattern (Q sort). They then completed a post‐sorting interview which provided insights into the reasons for participants’ placement of cards on the Q sort grid.^21^ Participants were given the option of continuing onto the demographic questionnaire and semi‐structured interview questions immediately after the post‐sorting interview or at a later time. The semi‐structured interviews provided information about residents’ views on prioritization and their experiences of unmet priorities. The first author (KL), who is experienced in conducting interviews with aged care residents, conducted the card‐sorting activity and interviews. Participants’ responses were audio recorded and transcribed, and photographs of participants’ completed Q sorts were taken. KL took field notes at the end of each study session about participants’ non‐verbal behaviours and their pace/ease of card sorting, as well as any interviewer reflections on patterns in the data.

#### 2.4.1 Analysis: Q methodology (research questions 1 and 2)

Q sort data were analysed using established techniques based on inverted factor analysis.^22^, ^23^ Specifically, centroid analysis and varimax rotation were used via PQMethod V.2.35.^24^ This analysis resulted in the identification of factors that represented shared meaning between groups of participants.^25^, ^26^ To determine the number of factors retained, the following criteria were used: the factor solution accounted for the greatest amount of variance explained while maximizing the number of Q sorts significantly loading on (ie correlating with) a single factor (factor loading ≥0.48, *P* < .01); each factor had an eigenvalue greater than 1; and two or more Q sorts significantly loaded on a factor.^25^, ^27^ PQMethod was used to produce a representative Q sort for each factor, known as a factor array (S1). Factor arrays were calculated as a weighted average of Q sorts loading on a particular factor.^26^

The viewpoints that factors represented were interpreted and presented as narrative accounts using participant transcripts, field notes, visual representations of factor arrays and crib sheets.^28^ Qualitative data were organized using NVivo V.12.^29^ Numerical factor array rankings were transformed into colour‐coded visual representations that classified cards by type of care (S2‐S5). This classification system was developed by KL through mind mapping techniques^30^ in which related cards were grouped together under five categories (Table 1). While some cards fit into multiple categories, cards were assigned to the most relevant category. This activity was guided by reviewing Australia's Accreditation Standards^31^ and aged care literature. Crib sheets summarized cards at ranks +3 and +4, distinguishing statements (cards ranked significantly different on one factor compared to others) and consensus statements (cards that do not significantly distinguish between factors).

**TABLE 1:** Care type categories

Care category	Definition	Card examples
Clinical care	Care addressing residents’ medical needs	Medication management; Resident decision making
Activities of daily living	Assistance with residents’ routine personal care	Skin care; Toileting
Respect	The treatment of residents in ways that value them	Respect; Privacy
Psychosocial care	Social, psychological and emotional aspects of care	Emotional Support; Conversations
Independence and choice	A relative concept referring to residents’ ability to do things for themselves and make decisions about non‐clinical aspects of care (clinical decision making is covered under ‘Resident decision making’)	Independence; Choice about meals

#### 2.4.2 Analysis: Inductive content analysis (research question 3)

Data from the think‐aloud activity, post‐sorting interviews and semi‐structured interviews were analysed using inductive content analysis via NVivo V.12.^29^ A random sample of transcripts (16%) were open coded by KL. Guided by Elo and Kyngäs,^32^ similar codes were grouped together under ‘generic categories’. These were further refined as ‘main categories’ which represented influences on prioritization decision making. This information was developed into an analytic framework by KL and KC. KL then analysed all transcripts using the analytic framework. The reporting of this data was guided by the Consolidated criteria for reporting qualitative studies (COREQ) checklist (S6).

## 3 RESULTS

### 3.1 Participant demographics

Thirty‐eight residents participated in the study, with 35 participants completing all aspects of the study. Three participants opted out of the semi‐structured interviews due to time limitations. Total study session times ranged from 14 minutes to 1 hour, 40 minutes (median = 40 minutes). Five participants had been interviewed by KL for an unrelated study 2 years earlier. For the other participants, no prior relationships existed. Participants completed the study either in their private room (n = 36) or a communal area (n = 2) with other residents and staff present. Three participants had a spouse (also a resident) present during the study. Sixteen additional residents were invited to participate in the study but did not take part due to inability to provide informed consent (n = 5), unavailability (n = 2), illness (n = 1), temporary residency at the facility (n = 1) or no reason given (n = 4).

The majority of participants were female (65.8%), 34.2% had been living in their current RACF for 1‐3 years, and 73.6% self‐rated their health as ‘Good’ or ‘Excellent’. Participants’ ages ranged from 72 to 97 years (median = 87.6 years), with the majority aged between 85 and 94 years (60.5%) (Table 2). Participants represented a variety of residents in terms of mobility, dependency, sensory functioning and medical conditions.

**TABLE 2:** Participant demographics

	n (%)
Age range	
<79	4 (10.5)
8‐84	7 (18.4)
85‐89	9 (23.7)
90‐94	14 (36.8)
>95	3 (8.0)
Not disclosed	1 (2.6)
Sex	
Male	13 (34.2)
Female	25 (65.8)
RACF location	
New South Wales	25 (65.8)
Queensland	13 (34.2)
Time living in facility	
<1 year	7 (18.4)
1 year‐2 years, 11 months	13 (34.2)
3 years‐4 years, 11 months	6 (15.8)
5 years‐6 years, 11 months	8 (21.1)
>7 years+	4 (10.5)
Self‐rated health	
Poor	3 (7.9)
Fair	7 (18.4)
Good	16 (42.1)
Very good	11 (28.9)
Excellent	1 (2.6)

### 3.2 Four‐factor solution

A four‐factor solution accounted for 54% of study variance and 31 Q sorts. The other seven Q sorts did not significantly load on any factor. Some of the factors were significantly correlated (Table 3), however, an examination of participant data revealed that the four factors represented different viewpoints: (a) maintaining a sense of spirituality and self in residential care, (b) information sharing and family involvement, (c) self‐reliance and (d) timely access to support. Participants’ transcripts provided context and nuanced meaning for each viewpoint. For example, participants may have ranked certain cards at the same level of importance across multiple factors, but participants’ interpretation of the cards and the reasons for their placement differed between factors.

**TABLE 3:** Correlations between factors

	Factor 1	Factor 2	Factor 3	Factor 4
Factor 1	1.0000	.4605	.6183*	.6675*
Factor 2	.4605	1.0000	.3101	.5332*
Factor 3	.6183*	.3101	1.0000	.5014*
Factor 4	.6675*	.5332*	.5014*	1.0000

* Significantly correlated at *P* < .01.

### 3.3 Viewpoints

Presented below are narrative accounts of each viewpoint on care prioritization. Single quotations represent card names, followed by factor array ranking in brackets. Single and double asterisks signify distinguishing statements at *P* < .05 and *P* < .01, respectively.

#### 3.3.1 Viewpoint 1: Maintaining a sense of spirituality and self in residential care

Viewpoint 1 accounted for 18% of variance and represented 10 Q sorts. Viewpoint 1 was characterized by the prioritization of ‘Spiritual activities’ (+4**) (S2), with most participants discussing the importance of religion in their lives. They valued opportunities to engage in spiritual activities, including the ability to attend on‐site daily mass, or walk to a nearby church. Participant 2 said:

With the Catholic church right next door, that’s important to me. That’s number one as far as I’m concerned.

Participants described themselves as being highly ‘Independent’ (+4). They spoke about managing their own care where possible, and speaking up when their needs weren't met. They also discussed the importance of being able to leave the facility when they wanted to. For some, the transition from independent living to a RACF was difficult, particularly in terms of loss of independence and privacy, as illustrated by the following quote from Participant 19:

If you said to me, what’s the hardest thing about coming into care? Loss of independence and privacy would feature high.

Participants were in agreement that ‘Privacy’ (+3*) was important. Although some said that their privacy was respected, others spoke about feeling disrespected by staff on occasion. The most commonly reported privacy‐related problem was staff entering residents’ rooms or bathrooms without knocking and waiting for an answer. Participant 15 shared the following:

Well some of them, they knock, they push the door and walk in. I told them, “Don’t walk in like that,” I said. “Sometimes I’m not dressed.” … Once when a fellow did that, I got angry with him. I said, “don’t do this … because I am a woman.”

The majority of participants loading on this viewpoint expressed dissatisfaction with food in terms of ‘Nutrition’ (+3**), appropriateness for older adults, taste, texture, the way food was prepared and ‘Meal choice’ (+2). Participants discussed the difficulties they experienced adjusting to the meals provided in RAC. Participant 25 commented:

It’s been a very important issue since I first came here. I was very disillusioned when I saw the meals and I thought, oh my God, I’ve eaten beautiful meals all of my life and I’ve been so aware of nutrition and fresh food and cooking properly, giving the correct meals to my family, and then I come in here and eat rubbish, absolute rubbish, really not very good food.

#### 3.3.2 Viewpoint 2: Information sharing and family involvement

Viewpoint 2 accounted for 17% of study variance and comprised 12 Q sorts. Participants loading on this viewpoint prioritized information sharing, specifically, ‘Family information’ (+4**) and ‘Resident information’ (+4) (S3). Residents explained that while they wanted to be informed about their medical care, it was more important that their family members were informed about, and involved in, their care. Participant 37 spoke about the importance of their daughter:

[My daughter] is everything to me, and she does everything for me, looks after my investments … and she does look after me … She’s my decision‐maker …

One explanation for this reliance on family is that residents loading on this viewpoint were dependent on other people for certain aspects of care. This was particularly true in their prioritization of ‘Bathing/showering’ (+2**) and ‘Assistance getting dressed’ (+1**). Many of the participants described being limited in their ‘Mobility’ (+1). They spoke about being ‘wobbly’ or prone to falls and as a result, needed wheelchairs, walking frames, assistance with ‘Repositioning’ (0**) or ‘Assistance with walking’ (−1). Despite this dependency, participants still valued their ‘Independence’ (+1), although this was ranked lower in Viewpoint 2 compared to participants associated with other viewpoints. When asked why independence wasn't ranked higher on the Q sort grid, Participant 8 responded:

Not the most important because I have to depend on other people to do things now.

Viewpoint 2 was also characterized by the low prioritization of choice‐related cards, for example ‘Seating choice’ (−4**), ‘Clothing choice’ (−3**) and ‘Choice about room environment’ (−2**). Participants explained that having choice was not a high priority, either because they were satisfied with the degree of choice available, or they were indifferent.

#### 3.3.3 Viewpoint 3: Self‐reliance

Viewpoint 3 accounted for 8% of study variance and represented five Q sorts. Similar to Viewpoint 1, ‘Independence’ (+4) was ranked as one of participants’ highest priorities (S4). For participants’ loading on Viewpoint 3, independence was conceptualized as being self‐reliant. When care staff were delayed in delivering care, some participants noted that they completed care activities without assistance. This group of participants prioritized their involvement in ‘Decision making’ (+3) as well as being keeping informed about their care (‘Resident information; + 2).

Participants’ self‐reliance was exemplified by the lower prioritization of ‘Family information’ (−1**) and ‘Attitudes towards family’ (−2**). For some participants, this was because their family members were no longer alive, or did not live close by. Others did not want their family members to be highly involved in their care, as illustrated by Participant 7:

Everybody feels or thinks that family is very important. Well I don’t because they have their own business, they have their own families etc and I’m just in the way. That’s why I came to [the facility] … so I can unburden them.

Participants communicated a preference for more individual‐based leisure activities, for example, reading or doing jigsaw puzzles. This could explain why ‘Choice about room environment’ (+3**) was ranked as one of participants’ highest priorities. As Participant 10 explained:

I do a lot of knitting here [in my room]. I do a lot of reading.

‘Privacy’ (+2*) was also a high priority, reflected in participants’ portrayal of themselves as being private people who liked to spend time alone. For example, Participant 7 stated:

I like my privacy. I make my own bed and I do everything. They [staff members] don’t even come into my room—just to give my medication and all that—but I like being alone …

#### 3.3.4 Viewpoint 4: Timely access to support

Viewpoint 4 accounted for 11% of study variance and comprised four Q sorts. Participants loading on this viewpoint were characterized by their preference for timely access to support from staff, particularly in terms of clinical support (eg ‘Medical conditions managed’; +4), ‘Call bell’ (+4**) and ’Emotional support’ (+2) (See S5).

While participants expressed a sense of urgency regarding the need for support, they acknowledged that staff members were often busy and therefore could be delayed in answering call bells. Participant 32 explained why they believed waiting for a call bell response was not appropriate:

If you ring your bell and it’s 10–15 minutes, that’s far too long. Because you don’t ring your bell unless you want something …

The importance of having staff member support extended beyond physical care to ‘Emotional support’ (+2), which was ranked highest in Viewpoint 4. When discussing the importance of emotional support, Participant 38 described a specific incident in which a visiting GP caused emotional distress. The participant expressed appreciation of the support they received from care staff:

In fact, one of the AINs [Assistants in Nursing] put in a complaint about her [visiting doctor] not respecting me. I was so upset I was in tears. The RN [Registered Nurse], she was wonderful.

Viewpoint 4 was also characterized by a lower prioritization of ‘Social activities’ (−3**), with participants explaining that they were satisfied with the availability of activities but often preferred to spend time alone, or socialize with their friends/family instead of engaging in organized group activities, as illustrated by Participant 32’s response:

I don’t attend many [social activities] as I’m a big reader… I generally just socialise around, talking to people or whatever, but I’m not a ‘craft’ person or anything like that …

### 3.4 Consensus statements

Consensus statements that were non‐significant at the *P* > .01 level (ie cards that did not distinguish between any two factors) included the following: ‘Monitoring/Safety’, ‘Mobility’, ‘Respect’, ‘Oral care’ and ‘Medical condition management’. The latter two were also non‐significant at the *P* > .05 level (S1).

Across the four viewpoints, clinical care, particularly management of residents’ medical conditions, was a high priority. Participants explained that their medical conditions often dictated the care that they needed in terms of assistance and medication. For some, medical management was seen as the primary reason they lived in a RACF. Participants also communicated that respect was a high priority. When asked why respect was important, Participant 36 said:

I think we have to realise that every person has dignity. And their dignity is respected and they’re not treated like animals or being abused or, you know, yelled at or whatever.

#### 3.4.1 Additional aspects of care

Box 1 outlines additional aspects of care that participants identified as not being well represented by the Q sort deck. Apart from palliative care and personal interests/entertainment, all other aspects of care were those that participants felt were inadequate (eg not enough staff training), or were related to prior negative experiences (eg loss of clothing through laundry services).

Box 1 **Additional aspects of care**

- Agency staff* (knowledge of care tasks and of residents, and attitudes towards caring)
- Cleanliness* (rooms, bathrooms and kitchen crockery)
- Communication about activities* (eg social outings)
- Laundry services and personal care of clothing*
- Maintenance of common areas
- Palliative care
- Personal interests/entertainment (eg card games, reading, TV)
- Staff members’ ability to effectively communicate in English* (ie communication breakdown between staff and residents)
- Staff members who listen to residents
- Staff training/experience/education*
- The taste of food
- The transition to institutional living
- Utilization of space*

*Suggested by multiple participants.

### 3.5 Influences on prioritization decision making

Across all participants, four influences on prioritization decision making were identified. These were labelled: (a) level of dependency; (b) dynamic needs; (c) indifference; and (d) availability of staff.

### 3.6 Level of dependency

Tasks that could be completed independently, without the assistance of staff members, were often given a lower priority. Common responses included variations of ‘I do that myself’, ‘I look after myself’ and ‘I don't need that’. Conversely, activities that required assistance were prioritized. For example, Participant 14 said:

I need to be showered each morning. Because I can’t do it myself. And then they assist me to dress.

Regardless of level of assistance needed, participants tended to prioritize ‘Independence’. They described wanting to try to ‘hold onto’ their independence for as long as possible, in whatever ways they could. Participant 24 explained how independence can operate in residential living:

Part of the problem for the old folk who come in here is … they feel they have lost their independence. But even when you have lost your independence and come to a place like this you can still have some independence. I mean, you can close the bloomin’ door and do what you like, and choose to go out on the balcony or not go out on the balcony. It’s a different kind of independence but it’s tremendously important …

### 3.7 Dynamic needs

Many participants spoke about their transition into residential aged care, including how their needs had changed over time. This transition involved adjusting to food, privacy, routines and room environment (ie reduction of living space). Participant 19 explained why room environment was important to them:

To establish myself because I have not yet called this place home, but I need to have a sense that this room is my place. And so, I needed to have important pictures on the wall, photos, I needed to have a bookcase, I needed things that made this room my own.

Participants would often use phrases such as ‘not yet’, ‘at this stage’ and ‘at the moment’, indicating that they were aware that their needs could change. Participants explained that things that were currently irrelevant or of little consequence might become more important. Participant 26 said:

I’m looking to the future a bit … I’m alright now, but if say, I live another couple of years, I’ve noticed that my health was not what it was three years ago.

### 3.8 Indifference

Participants sometimes expressed indifference towards some aspects of care. They spoke about not being ‘fussed’, ‘bothered’, ‘worried’ or ‘interested’ in response to certain cards, assigning them a lower priority. For example, Participant 31 said:

I don’t care where I sit. I don’t care what’s in the [my] room.

An attitude of indifference was particularly relevant to some of the choice‐related cards. For some participants, this was because they did not mind whether they had choices or not. For others, this was because certain aspects of care were already occurring. The following response by Participant 31 illustrates this perspective:

It doesn’t really matter because I dress myself in the morning, I just pick the clothes I want and that’s it.

### 3.9 Availability of staff members

A recurring theme throughout participants’ responses was that there were not enough staff, in terms of overall numbers, their busyness and the number of permanent (vs agency) staff. Participants shared examples of when staff shortages had led to missed, rushed or delayed care, and unmet needs. Participant 13 explained that help was sometimes difficult to find:

You could turn around and say, ‘Where’s the carers? Where? Where? I want a carer. Where are they?’ You can’t get one, there’s no one around. And some [residents] have got buzzers, they could press their buzzers and nothing happens. As I say, they’ve [staff] got jobs, but then again they are supposed to be looking after me as well … how can they look after me if they’re down working somewhere else?

Two examples, ‘Conversations’ and ‘Call bell’ demonstrate how availability of staff influenced participants’ priorities in different ways. ‘Conversations’ was ranked as either a neutral or low priority across factors. Participant 15 explained that ‘Conversations’ was a lower priority, as staff members did not have the time to chat:

They don’t spend much time with you because they’re busy, busy, busy. When they’re chatting with you, somebody will press the buzzer [call bell].

‘Call bell’ was ranked as either a neutral or high priority across viewpoints. Although some participants said that their call bells were answered immediately, often because it was rare for them to ring their call bell, other participants communicated that they were left waiting. For some, like Participant 9, ‘Call bells’ was a high priority because they recognized the urgency of needing help:

Well I’ve had plenty of incidences. You know, they take at least an hour whenever you ring. And it’s not good enough, you know, really. You could be dead on the floor.

Other participants acknowledged that staff members were busy attending to other residents who might be in greater need and therefore understood they needed to ‘wait their turn’. Some participants also acknowledged that problems generated by inadequate staffing were an organizational or systems issue and not a reflection on front‐line staff. On the whole, participants spoke extremely highly of staff members, describing them as ‘kind’, ‘sweet’, ‘caring’, ‘friendly’, ‘patient’ and ‘supportive’. For example, Participant 9 said:

They’re [staff members] here to earn a living, but you know, some of them are absolutely wonderful … what they would do for you, if they had to. They are very friendly, and very nice, and go out of their way …

## 4 DISCUSSION

### 4.1 Residents’ priorities and prioritization of care

This study explored residents’ priorities for their care, how they prioritize care and influences on their prioritization decisions. Residents were able to identify their priorities and communicate why certain aspects of care were more or less important to them. Residents’ prioritization of care was found to be a reflection of their need for assistance, experiences, preferences and views about receiving support from others. While residents’ prioritization of care was based on individual circumstances, four overarching viewpoints on prioritization were identified: maintaining a sense of spirituality and self in residential care; information sharing and family involvement; self‐reliance; and timely access to support.

Across the four viewpoints, two common priorities emerged: being treated with respect by staff members and the management of medical conditions. Our findings are in accordance with Bangerter et al who found that one of residents’ highest preferences for care was staff members showing respect, reflected in staff members’ attitudes, communication, professionalism, etiquette, greetings, person‐directed care and reciprocity.^33^ Our findings also provide some evidence of alignment between residents’ and staff members’ priorities as ‘medical condition management’ and ‘respect’ were also high priorities for the majority of participants in our related study on staff members’ prioritization of care.^34^

While independence was not identified as a consensus statement, factor arrays and participant transcripts revealed that having independence in RACFs was important to residents regardless of which viewpoint they loaded on. Independence was found to mean different things for different residents. For example, participants loading on Viewpoint 1 conceptualized independence as a way of maintaining a sense of self after moving to a RACF. Aligning with previous research,^35^, ^36^ we found that loss of independence was particularly relevant to the transition period of moving from one's own home into a RACF. Participants loading on Viewpoint 3 conveyed that, to them, independence meant having the ability to make decisions about their care, as well as having a sense of being able to rely on themselves rather than others. Even residents who were more physically dependent, such as those loading on Viewpoint 2, valued their independence. Although residents depended on support in some aspects of their care, they sought ways in which to exercise their independence in other ways. This finding resonates with research from the UK^37^, ^38^ that found that independence was a relative term, as residents focused on minor daily accomplishments of autonomy. While our research revealed that having independence is important to aged care residents, a recent survey^39^ of Australian aged care employees found that 79% of residential aged care staff (n = 723) reported not having enough time to support residents to do things for themselves.

### 4.2 Influences on prioritization decision making

Participants made prioritization decisions based on the degree to which they needed assistance from others (level of dependency) and what they anticipated their needs might be in the future (dynamic needs). Participants acknowledged that their needs might change over time, especially in response to a decline in physical and cognitive functioning. Participants’ prioritization decisions were also found to be influenced by their personal preferences, reflected in their ‘indifference’ and lack of interest towards some aspects of care. Similar to our findings, Heid et al^40^ reported that ‘within‐person factors’, including functional dependency and residents’ level of interest, influenced residents’ perceived importance of their preferences. Heid et al^40^ also found that residents’ preferences were situational and were likely to change in response to personal and environment circumstances. Other influences identified by the authors were social factors (eg type of staff relationship), global factors (eg the weather) and facility environmental factors (eg policy and resources). While social and global influences on prioritization decisions were not revealed by our research, environmental factors, specifically labour resources, were found to influence participants’ decision making.

Participants conveyed that staff members appeared busy and rushed due to staffing shortages. We found that perceived lack of staff availability influenced participants’ prioritization decisions, particularly in relation to having conversations with staff members and call bells answered in a timely manner. ‘Conversations’ was often viewed as a lower priority, with residents explaining that staff members were often too busy to engage in meaningful conversation. Talking with residents has previously been identified as a commonly reported missed and rushed care activity in Canadian RACFs.^41^ Additionally, Meagher et al^39^ found that of the 946 residential aged care staff surveyed, 91% reported not having enough time to listen and connect with residents, and 84% reported not having enough time to talk with residents during mealtimes.

‘Call bells’ was seen as a higher priority for many participants as they described waiting long periods of time for a response from staff members, who were often busy helping other residents. This supports the finding by Meagher et al that 46% of residential aged care workers surveyed were either ‘always or often’ unable to respond to call bells within five minutes, with an additional 35% ‘sometimes’ unable to respond.^39^

### 4.3 Recommendations for policy and practice

In order to deliver care that aligns with residents’ priorities, we put forth the following recommendations for policy and practice:

1. *Encouraging open communication between staff members and residents regarding care priorities*. Residents in our study were able to identify their priorities and communicate why certain aspects of care were more or less important to them. Open and continuous communication channels with residents could help staff better understand residents’ priorities and how these might change over time. For residents with communication difficulties, an understanding of non‐verbal communication cues^42^ and seeking personal knowledge from family members^43^ could facilitate an understanding of residents’ priorities.
2. *Supporting residents’ independence*. Maintaining independence was important for participants, regardless of the viewpoint they loaded on. Independence may be restricted in RACFs due to routines, concerns over safety, and inadequate time to encourage residents to do things for themselves.^39^, ^44^, ^45^ Residents in our study identified several strategies that care staff could implement to facilitate independence, including:
  - Supporting residents to carry out tasks themselves, for example, allowing residents to shower themselves while supervising them.
  - Partially completing tasks while encouraging resident involvement, for example, putting on residents’ socks but letting them put on the rest of their clothes.
  - Flexibility of routines, for example, working around residents’ preferences for the timing of showering (morning vs night).
  - Respecting residents’ preferences, for example, letting them make their own bed if they want to.
3. *Ensuring safer staffing ratios*. Participants reported that perceived staffing shortages affected the way in which care was delivered and how they prioritized care. They expressed that staff members were doing their best, but were sometimes unavailable to provide care and often appeared rushed. The 2019 interim report from the Australian Royal Commission into Aged Care Quality and Safety identified that low staffing levels coupled with high workload pressures were contributing factors to substandard care. Furthermore, the Australian Nursing and Midwife Federation's 2016 MISSCARE survey^46^ found that of 3,206 RACF staff members surveyed, only 8.2% reported that staffing levels were ‘always’ adequate. Our findings, along with other published research and stakeholder reports,^46^, ^47^, ^48^ underline the importance of safer staffing levels in order to meet residents’ needs and priorities.

### 4.4 Strengths and limitations

The study design enabled residents with varying needs to participate in the study, including those with hearing loss, mobility issues, speech impairment, vision impairment and mild cognitive impairment. During the card‐sorting activity, cards could be manually sorted by participants or read out and placed on the board by the interviewer. The cards were tailored to meet the needs of older adults: large text was used, they were printed on thick cardboard to avoid skin cuts, and representative images were used to help residents easily identify cards.

The recruitment criteria excluded residents who were unable to give informed consent or those that may have been physically or emotionally burdened by the study. Consequently, the sample was biased towards residents who had higher cognitive capacity and physical health. As Q methodology can be a cognitively demanding tasks, we recommend that future studies include residents with cognitive impairment in studies of care prioritization using survey methods,^49^, ^50^ interviews^51^ or family proxies.^18^, ^52^ Despite this limitation, the sample comprised residents with a variety of needs, self‐perceived levels of health, medical conditions and functional abilities. While the sample was limited to a single provider, participants were recruited from five RACFs across two Australian States in an attempt to reduce the influence of environmental context.

Another limitation was that the study captured residents’ priorities at a single point in time. Participants acknowledged that their needs were dynamic and were expected to change in the future. To provide a more accurate representation of prioritization, longitudinal studies that map residents’ prioritization of care over time are needed.

## 5 CONCLUSIONS

Our study demonstrated that residents meeting the participant inclusion criteria were capable of prioritizing care and explaining their prioritization decisions. While different viewpoints on care prioritization were identified, participants across the sample prioritized being treated with respect, management of their medical conditions and their independence. Residents’ prioritization decisions were found to be influenced by personal factors (dependency, dynamic needs, level of interest) and environmental factors (staffing resources). The recommendations arising from this research are applicable to an international context, as residential aged care systems in developed countries face challenges to meetings residents’ priorities, similar to those identified by the present study, the Australian Nursing and Midwifery Federation and the Australian Royal Commission into Aged Care Quality and Safety.

## CONFLICTS OF INTEREST

The authors have no conflicts of interest to declare.

## AUTHOR CONTRIBUTIONS

KL conceptualised, designed and led the study. KC, VM, LAE and JB contributed to the study design. KL, KC, VM and LAE developed the study materials. KL recruited participants, and collected, analysed, and interpreted data. KC assisted with the interpretation of data. KL drafted the manuscript with all authors contributing to manuscript revisions. All authors approved the final submission.

## ETHICS APPROVAL AND INFORMED CONSENT

The study was developed in accordance with national ethics guidelines.^17^ It was approved by the Macquarie University Human Research Ethics Committee and the Human Research Ethics Committee of the participating aged care provider. Informed consent was obtained from all participants.

## PATIENT OR PUBLIC CONTRIBUTION

Individuals who had a parent residing in residential aged care piloted the card‐sorting activity to ensure that cards and the care they represented were appropriate for aged care residents in terms of imagery and language.

## Supporting information

Supplementary material S1‐S5

Supplementary material S6

## Data Availability

The data that support the findings of this study are available from the corresponding author upon reasonable request.
