# Supplementary material for: Aged care residents’ prioritization of care: A mixed‐methods study
Source: Health Expect. 2021 Jan 21;24(2):525–36. doi: 10.1111/hex.13195 (PMC8077118; doi:10.1111/hex.13195)
Supplement: Supplementary file 1 — Supplementary material S1‐S5 [file HEX-24-525-s001.docx]

**S1: Q cards, Q statements, care categories and factor arrays**

| **Card label** | **Q Statement** | **Care category** | **Factor arrays: card rankings** | | |  |
| --- | --- | --- | --- | --- | --- | --- |
|  |  |  | **Factor 1** | **Factor 2** | **Factor 3** | **Factor 4** |
| Assistance getting dressed | Assistance getting dressed when needed | Activities of daily living | -3 | +1** | -2 | -2 |
| Assistance with meals | Assistance with meals when needed | Activities of daily living | -2 | -3 | -2 | -4 |
| Assistance with walking | Assistance with walking when needed | Activities of daily living | -2 | -1 | -1 | -3 |
| Attitudes towards family | The facility/home is welcoming to family members | Psychosocial care | +1 | 0 | -2** | 0 |
| Bathing and showering | Assistance with bathing/showering when needed | Activities of daily living | -2* | +2** | 0** | -4* |
| Bowel care | Bowel care is provided when needed | Activities of daily living | -1** | +1 | -4** | +1 |
| Call bell | Call bell is responded to in a timely manner | Clinical care | +1 | +1 | 0 | +4** |
| Choice about room environment | Choice about what is in my room | Independence and choice | 0 | -2** | +3** | 0 |
| Clothing changed | Clothes are changed when needed | Activities of daily living | -1 | -1* | -2 | -2 |
| Clothing choice | Choice about the clothes I wear | Independence and choice | -1 | -3** | +1** | -1 |
| Conversations | Carers/nurses chat with me | Psychosocial care | 0 | -3* | 0 | -1 |
| Emotional support | Emotionally supported by carers/nurses | Psychosocial care | 0 | 0 | +1 | +2 |
| Family information | My family is informed about my medical care | Clinical care | +1 | +4** | -1** | +1 |
| Independence | I have independence | Independence and choice | +4 | +1 | +4 | +2 |
| Meal choice | Choice about meals | Independence and choice | +2 | -2 | -1 | +1 |
| **Medical condition management** | **Medical conditions are managed** | **Clinical care** | **+2** | **+3** | **+3** | **+4** |
| Medication management | Correct medication at the right time | Clinical care | +2 | +3 | +2 | +3 |
| **Mobility** | **Supported to keep active and mobile** | **Clinical care** | **+1** | **+1** | **0** | **+2** |
| **Monitoring/ Safety** | **I am kept safe from injury or medical harm** | **Clinical care** | **+1** | **+2** | **+1** | **0** |
| Nail care | Nail care is provided when needed | Activities of daily living | -3** | -4** | 0 | -1 |
| Nutrition | Meals are nutritious | Clinical care | +3** | -2** | +1 | +1 |
| **Oral care** | **Regular dental and oral care** | **Activities of daily living** | **-1** | **-2** | **-1** | **0** |
| Personal grooming | Assistance with personal grooming when needed | Activities of daily living | -3 | -1* | -3 | -2 |
| Privacy | My privacy is respected | Respect | +3* | 0* | +2* | -1* |
| Repositioning | Assistance with repositioning when needed | Activities of daily living | -4 | 0** | -4 | -3 |
| Resident decision-making | Involvement in making decisions about my care | Clinical care | 0* | +2 | +3 | +3 |
| Resident information | I am informed about my medical care | Clinical care | 0** | +4 | +2 | +3 |
| **Respect** | **Care staff treat me with respect** | **Respect** | **+3** | **+2** | **+2** | **+2** |
| Seating choice | I can choose where to sit during group activities | Independence and choice | -1 | -4** | +1** | -2 |
| Skin care | Skin care is provided when needed | Activities of daily living | -2 | -1 | -3 | 0* |
| Social activities | Variety of social and leisure activities offered | Psychosocial care | 0 | 0 | 0 | -3** |
| Spiritual activities | Opportunities to engage in spiritual activities | Psychosocial care | +4** | 0 | -1 | 0 |
| Staff knowledge | Staff are knowledgeable about my medical care | Clinical care | +2 | +3 | +4 | +1 |
| Toileting | Assistance with toileting needs | Activities of daily living | -4 | -1 | -3 | -1 |

***** Distinguishing statement at p<0.05

** Distinguishing statement at p<0.01

Consensus statements at p>0.01 are bolded

**S2: Visual representation of Factor 1 factor array**

**Legend**

Respect

Clinical care

Activities of daily living

Psychosocial care

Independence and choice

| **-4** | **-3** | **-2** | **-1** | **0** | **+1** | **+2** | **+3** | **+4** |
| --- | --- | --- | --- | --- | --- | --- | --- | --- |
| Assistance with repositioning | Assistance getting dressed | Skin care | Clothing changed | Resident decision-making | Call bell | Medical condition management | Nutrition | Spiritual activities |
| Toileting | Nail care | Assistance with walking | Oral care | Resident information | Monitoring/ Safety | Staff knowledge | Respect | Independence |
|  | Personal grooming | Bathing/ Showering | Bowel care | Emotional support | Family information | Medication management | Privacy |  |
|  |  | Assistance with meals | Seating choice | Conversations | Mobility | Meal choice |  |  |
|  |  |  | Clothing choice | Social activities | Attitudes towards family |  |  |  |
|  |  |  |  | Choice about room environment |  |  |  |  |

**S3: Visual representation of Factor 2 factor array**

| **-4** | **-3** | **-2** | **-1** | **0** | **+1** | **+2** | **+3** | **+4** |
| --- | --- | --- | --- | --- | --- | --- | --- | --- |
| Nail care | Assistance with meals | Nutrition | Clothing changed | Assistance with repositioning | Call bell | Monitoring/ Safety | Medical condition management | Family information |
| Seating choice | Conversations | Oral care | Skin care | Emotional support | Mobility | Resident decision-making | Staff knowledge | Resident information |
|  | Clothing choice | Meal choice | Assistance with walking | Social activities | Bowel care | Bathing/ Showering | Medication management |  |
|  | **Legend**  Respect  Clinical care  Activities of daily living  Psychosocial care  Independence and choice | Choice about room environment | Personal grooming | Spiritual activities | Assistance getting dressed | Respect |  |  |
|  |  |  | Toileting | Attitudes towards family | Independence |  |  |  |
|  |  |  |  | Privacy |  |  |  |  |

**S4: Visual representation of Factor 3 factor array**

| **-4** | **-3** | **-2** | **-1** | **0** | **+1** | **+2** | **+3** | **+4** |
| --- | --- | --- | --- | --- | --- | --- | --- | --- |
| Assistance with repositioning | Skin care | Clothing changed | Family information | Mobility | Monitoring/ Safety | Medication management | Medical condition management | Staff knowledge |
| Bowel care | Personal grooming | Assistance getting dressed | Oral care | Call bell | Nutrition | Resident information | Resident decision-making | Independence |
|  | Toileting | Assistance with meals | Assistance with walking | Nail care | Emotional support | Respect | Choice about room environment |  |
|  | **Legend**  Respect  Clinical care  Activities of daily living  Psychosocial care  Independence and choice | Attitudes towards family | Spiritual activities | Bathing/ Showering | Clothing choice | Privacy |  |  |
|  |  |  | Meal choice | Social activities | Seating choice |  |  |  |
|  |  |  |  | Conversations |  |  |  |  |

**S5: Visual representation of Factor 4 factor array**

| **-4** | **-3** | **-2** | **-1** | **0** | **+1** | **+2** | **+3** | **+4** |
| --- | --- | --- | --- | --- | --- | --- | --- | --- |
| Bathing/ Showering | Assistance with repositioning | Clothing changed | Toileting | Monitoring/ Safety | Family information | Mobility | Resident decision-making | Call bell |
| Assistance with meals | Assistance with walking | Assistance getting dressed | Nail care | Oral care | Staff knowledge | Emotional support | Medication management | Medical condition management |
|  | Social activities | Personal grooming | Conversations | Skin care | Nutrition | Independence | Resident information |  |
|  | **Legend**  Respect  Clinical care  Activities of daily living  Psychosocial care  Independence and choice | Seating choice | Clothing choice | Spiritual activities | Bowel care | Respect |  |  |
|  |  |  | Privacy | Attitudes towards family | Meal choice |  |  |  |
|  |  |  |  | Choice about room environment |  |  |  |  |
